# On Pyrogen, or the Electric Fluid

**Published:** 1848-06

**Authors:** John Joseph Lake

**Affiliations:** Portsmouth


*On Pyrogen, or the Electric Fluid.*—By JOHN JOSEPH LAKE, Esq., Portsmouth.—The property of ponderability in pyrogen, developed in the experiment detailed in my communication published in the Lancet of the 12th January last, is only to be observed under peculiar circumstances, it being commonly concealed by the operation of other more powerful influences.

The most potent of all is the property of repulsion existing between its particles. These, acting upon the mechanical principle that bodies when within a certain distance repel each other, continually endeavour to divide; and this action is so intense, that when a body (as a sphere) is charged with the fluid, it remains entirely on the exterior; even on a roll of gauze wire it takes it place solely on the outer surfaces. Now our globe is such a sphere powerfully charged, and on this principle the fluid should extend itself as high as possible, and its greatest density should be at the point most remote from the centre of the earth. This is actually found to be the case. Mr. Sturgeon, in detailing his ingenious experiments, says, "Every vertical column of a dry, cloudless atmosphere, whatever may be its dimensions, is constantly electropolar in one and the same direction, having its positive pole upwards; "I have made more than five hundred experiments with kites, for exploring the electricity of the atmosphere, and in every case where clouds do not interfere I have found the upper strata positively electrical with reference to those that are below. I have had three kites, and sometimes five, at different altitudes at the same time, and have transmitted sparks from one to another from the top to the bottom of the series; in every case I found the uppermost of a pair to be the positively electrized stratum of the atmosphere.

Hence we may see the reason that the ponderability of pyrogen has hitherto escaped attention. Its general tendencies are quite in another direction, not because it is not attracted by the earth, but because the force with which its particles endeavour to separate is such that the fluid seeks a larger circumference over which to diffuse itself, and this principle of separation is seen to be so intense, that the pyrogen even rises into a non-conducting medium to give way to it.

This obedience of the particles of pyrogen to a purely mechanical law affords another most convincing proof of its materiality.

There are two other influences to be noticed as greatly increasing this upward tendency—namely, the centrifugal force resulting from the earth's motion on its axis, and the attraction of the sun. The first requires no explanation. As to the second, the sun by its attractive power keeps the earth in its orbit, and attracts the pyrogen, as well as the other matter of which it is composed, which, in my opinion, is the main cause of the magnetic tides; for the earth in its daily revolutions leaves a pyrogenic or electric wave constantly raised on the side presented to the sun.

A third deduction may be made from the experiment detailed in my former paper, (the Lancet, Jan. 15th,)—namely, that light is developed during the passage of pyrogen through the atmosphere: this, coupled with the preceding remarks, leads to some important consequences.

It has been satisfactorily proved by Mr. Faraday, that pyrogen circulates continually about the earth, and by taking advantage of this discovery electric clocks have been constructed. In this circulation light must be developed, and this is, in my opinion, the cause of the luminosity of our planet, so frequently seen at night, a phenomenon that has also been noticed at times in Venus. And shall we not ascend a little higher and apply these laws to the sun, with its denser atmosphere, under the pressure of which it is probable that carbonic acid ceases to be a gas, and other bodies, volatile or fluid with us, assume the form of liquids or solids? There we have evidences of similar effects. The circulation of the fluid on that sphere being more rapid, and through a denser medium than with us, exhibits a greater intensity of light, as it has a greater opposition to overcome in its passage; hence the intensity of its light, and seeking, as on the earth, the higher regions of the atmosphere, yet controlled by the centrifugal force resulting from the sun's motion on its axis, and the attractions of the planets, it produces the zodiacal lights.

The above theories accord equally well with the phenomena observed on the moon, in which the atmosphere should be more rare than that of the earth, on account of its inferior size, and which, in fact, is found to be so rare that astronomers doubt whether there is any at all. Now, since the moon only revolves on its axis once a month, the pyrogenic or electric currents must be very slow, and utterly insufficient to carry on the various chemical compositions and decompositions necessary for the support of the vegetable world; and no signs of vegetation have been discovered upon its surface, although Lord Rosse's telescope is of sufficient power for the purpose, if grass or anything of the kind existed there, since it would give a green colour to the soil, whilst it is observed to be of a brownish hue. This dull movement of the fluid also accounts for the absence of self-luminosity in the moon; for the atmosphere being in addition very rare, the fluid has little or no opposition to overcome.

The above phenomena are all that I shall at present bring forward of that kind in evidence of the materiality of pyrogen. I will close with a few remarks on some of its other properties.

When electric or pyrogenic sparks are passed through a medium of atmospheric air, nitric acid is formed by the union of portions of its nitrogen and oxygen. Therefore, during the circulation of this body about our globe, as above referred to, nitric acid must be formed, for the light generated is the result of a series of infinitely small sparks, which arise from air being a non-conductor. If the fluid move by a good conductor, as undistilled or acidulated water, no light would be developed. Hence the origin of spontaneous nitrification and the saltpetre earths of India and other countries; for nitric acid, being produced in this manner, seizes upon the potash of the decaying vegetable world, and forms nitrate of potash, or saltpetre.

I have shown, in the papers referred to in my former communication, that pyrogen is not only the *begetter* of fire, but also of acids and oxides, or, at least, of many, together with a large amount of the chemical combinations that are continually taking place in the animal and vegetable world. In this respect some of its effects are analogous to those of water on certain mixtures, as, for instance, on proportions of tartaric acid and carbonate of soda, which may be mixed together in a dry state without acting on each other; although, if a little water be added, effervescence takes place and new combinations result—e. g., the case of potassium. This was first obtained by negatively electrifying moistened hydrate of potash; that is, by withdrawing pyrogen from it. The affinity between the metal and oxygen is thus destroyed, and the former appears. As soon as the communication with the battery is broken, and the metal exposed to the air, it tarnishes, and is quickly covered with a crust of caustic potash; for pyrogen immediately returns and restores the affinity between the metal and oxygen, which is sufficiently strong to absorb the gas from the atmosphere. In a similar manner, though with a more violent operation, it oxidizes when thrown upon water.

Sodium, the ammoniacal amalgam, lithium, barium, strontium, and other similar oxidizable metals, afford illustrations of the operation of the same principle. The flame observed in some instances, arises from the union of the released hydrogen of the water and the oxygen of the air, promoted by the presence and rapid development of pyrogen, in the way I have shown in explaining the causes of the ignition of metals in acid solutions.—*Ibid.*

